# Flexural performance of reinforced concrete beams exposed to uncontrolled fire with and without plaster protection

**DOI:** 10.1038/s41598-025-00146-8

**Published:** 2025-08-26

**Authors:** Naila Hisbani, Muhammad Alamgeer Shams, Mohammad Ajmal Stanikzai, Hamad Almujibah, Omrane Benjeddou

**Affiliations:** 1 Civil Engineering Department, University of Technology Petronas, Ipoh, Malaysia; 2 Civil Engineering Department, Nangarhar University, Jalalabad, Afghanistan; 3 Department of Civil Engineering, Taif University, Taif, Saudi Arabia; 4 Department of Civil Engineering, Prince Sattam bin Abdulaziz University, Al-Kharj, Saudi Arabia

**Keywords:** Fire test, Reinforced concrete beam, Fire response, Flexural behaviour, Plaster effect, Mechanical properties, Composites

## Abstract

Fire analysis of reinforced concrete (RC) structures is vital for understanding their behavior under extreme conditions. In real-world scenarios, uncontrolled fires can damage buildings and infrastructure substantially, undermining their structural integrity. Assessing the performance of RC beams under fire exposure is crucial for ensuring the safety and reliability of structures. This study examines the structural performance and residual moment capacity of plastered and unplaster-reinforced concrete (RC) beams subjected to varying durations of fire exposure. Two types of reinforcement configurations in plastered and unplaster beams were evaluated: one with two 10 mm diameter reinforcement bars at the top and bottom and another with two 10 mm diameter bars at the top and three at the bottom. Unplaster and plastered beams were made from concrete with a compressive strength of 20 MPa at ambient temperature. They were exposed to fire for durations of 0, 3, and 6 h, simulating real fire conditions with a peak temperature of 800 °C. Beams exposed to fire showed reductions in moment capacity of about 20–36%, depending on the reinforcement. The findings highlight the importance of considering plaster as a protective layer in RC beam design to enhance fire resistance and post-fire structural performance. The findings emphasize the necessity of incorporating fire-resistant measures, such as plaster coatings, to mitigate structural degradation, thereby enhancing post-fire safety and performance of RC beams.

## Introduction

The term “fire” often evokes feelings of unease, conjuring potential threats to both human life and economic stability. Structural damage caused by fires can vary from superficial discoloration to complete collapse, posing serious risks. Ensuring safety demands a proactive approach that encompasses engineering solutions, public education, and the strict enforcement of fire safety measures. These precautions, rooted in centuries of human experience with fire, have consistently proven effective in reducing the frequency and severity of fire-related accidents. Concrete structures are rapidly emerging around the globe. Concrete is a heterogeneous material composed of aggregates, a binder, and steel or other forms of reinforcement. Each material has a distinct composition and exhibits different behaviors when exposed to fire. In other words, the behavior of reinforced concrete materials is related to temperature-dependent material properties^1^. Concrete is regarded as a fire-resistant material due to its very low thermal conductivity, while steel is quite the opposite in terms of manufacturing and thermal properties^2^. Reinforced concrete (RC) structures have been the focus of studies at various levels, including frame structures such as beams and columns^3–6^. Studies on beams subjected to fire, which are highly relevant to this study, are reviewed below. Gao et al.^7^ conducted a numerical heat analysis using FEM models to predict the fire response of reinforced concrete beams. The 3D finite element (FE) model enables a detailed analysis of the local behavior of reinforced concrete (RC) beams under fire conditions, capturing stress and deformation states in both the concrete and steel and their intricate interaction. This proposed 3D FE model is a cost-effective numerical tool for the performance-based fire safety design of RC beams^7^. In another study^8^, beams were exposed to fire and retrofitted using a furnace within a controlled lab environment. Different parameters, such as fire duration, type of fire exposure, and FRP strengthening of the beam, were considered as the main factors in the study. In the study, efforts were made to produce real-time fire scenarios even with the application of the load and boundary conditions. However, the study used propane burners as well to maintain the temperature-time curve as per the standard temperature vs. time curve^8^. Similar recent studies on the fire exposure of reinforced concrete beams—such as those by Cai et al.^9^ and Thongchom et al.^10^—used numerical or finite element methods to predict the post-fire moment capacity of the beams.

In other words, studies focusing on the thermal response of concrete have utilized a furnace or uniform heating and cooling methods^11–13^ to achieve high temperatures. Testing concrete structures against fire is costly and risky. To mitigate experimental costs and setup challenges, recent developments are employing numerical modeling to predict the post-fire response of concrete members^14,15^. A recent study compiling data from 216 fire-tested specimens identified key influencing parameters such as load ratio, concrete cover thickness, and reinforcement ratio and developed a fire resistance prediction model using machine learning^16^. These findings highlight the growing role of data-driven methods in fire safety assessment. Apart from fire resistance, recent studies have also investigated the repair of fire-damaged concrete members. The use of FRP composites has been shown to restore or enhance the axial capacity of fire-damaged RC columns^17^. Additionally, alternative strengthening methods, such as near-surface-mounted (NSM) rebars and ultra-high-performance fiber concrete (UHPFC) jacketing, have significantly improved the load-carrying capacity of heat-damaged RC columns^12^. These advanced strengthening techniques provide viable rehabilitation strategies for fire-affected structures. Fire performance studies have also explored prestressed concrete (PC) beams under combined fire and structural loading. Experimental research on PC beams exposed to both fire and load conditions revealed that fire-induced restraint forces marginally enhance fire resistance due to constrained thermal expansion^18^. Furthermore, numerical models incorporating thermal expansion, cracking, spalling, and geometric effects have been validated for predicting fire response in PC beams^18^. Most research has been conducted under controlled laboratory conditions with maintained fire temperatures, such as in a kiln or furnace. However, when an unexpected fire occurs in a building, there is no controlled fire temperature. Multiple factors contribute to the fire scenario, including wind and uncontrolled fire and the duration of the fire. Concrete members in the building and airflow around it provide fuel for the fire’s continuation. Considering the actual fire scenario, this study focuses on the experimental and numerical investigation of doubly reinforced concrete beams under fire, taking into account real-time fire phenomena. The second factor addressed in this study is the effect of reinforcement in the tension zone and the impact of shear reinforcement on the exposed reinforced concrete beam.

## Experimental methodology

### Design of RC beams

**Fig. 1:**
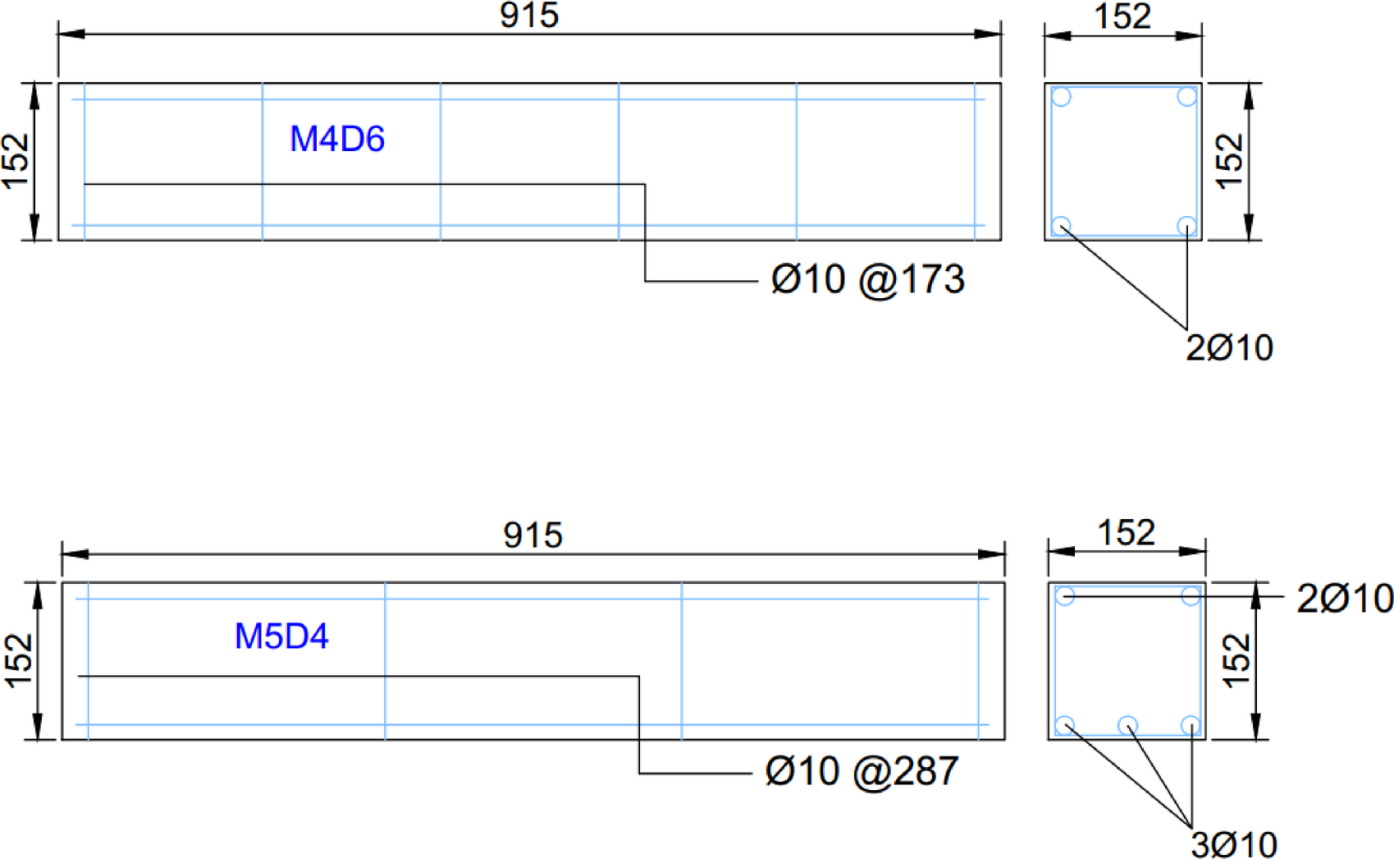
Detailing of specimen M4D6 and M5D4 (dimensions in mm).

**Fig. 2:**
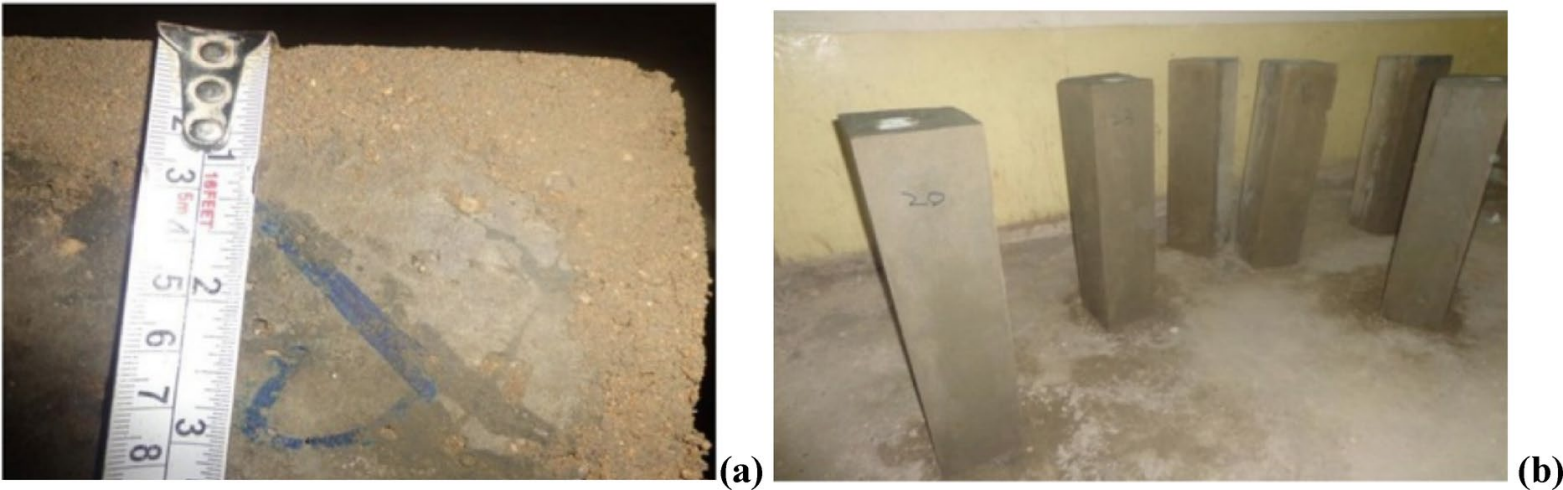
Plaster beams before testing, (**a**) thickness of plaster, (**b**) Plastered beams before fire exposure.

This study involved casting a total of 36 reinforced concrete beams to investigate the effects of fire exposure on their structural performance. The beams were divided into two main configurations based on their reinforcement detailing: (1) beams with 4 main bars and 6 stirrups, designated M4D6, and (2) beams with 5 main bars and 4 stirrups, referred to as M5D4. Each configuration consisted of 9 beams, which were subjected to fire exposure durations of 3 and 6 h. Within each group of 9 beams, 3 specimens were reserved as control samples that were not exposed to fire, serving as a baseline for comparison. To simulate real-world construction practices, an additional set of 9 beams for each configuration (M4D6 and M5D4) was prepared with a 20 mm thick plaster coating. These plastered beams were allowed to cure for 7 days after application, ensuring that the curing process reflected typical on-site conditions. The inclusion of plastered beams aimed to evaluate the potential protective effects of plaster under fire conditions, providing insights into its role in enhancing fire resistance. This experimental design enabled a comprehensive assessment of the influence of reinforcement detailing, fire exposure duration, and plaster application on the structural integrity of the beams, while maintaining a balance between scientific rigor and practical relevance. Table 1 below shows the specimen details used in this study, while Fig. 1 illustrates the schematic diagram of the reinforcement arrangements in the beams. The target strength of 20 MPa was achieved using a W: C:S: A ratio of 0.4:1:2:4. A lower grade of concrete was selected to obtain better effects of fire on the reinforced concrete beams. Figure 2 displays the beams produced for testing.

**Table 1:** Experimental details of the specimen used in testing.

Sr. #	Fire exposure time (hrs.)	Beam Designation	Steel Design	Description of Beam
1	0	M4D6	M.S* = 4, D. S*=6	#10 diameter rebar for main steel and distribution steel.
2	3	M4D6-3	M.S* = 4, D. S*=6	
3	6	M4D6-6	M.S* = 4, D. S*=6	
4	0	M5D4	M.S* = 5, D. S*=4	
5	3	M5D4-3	M.S* = 5, D. S*=4	
6	6	M5D4-6	M.S* = 5, D. S*=4	
7	0	M4D6-P	M.S* = 4, D. S*=6	
8	3	M4D6-P3	M.S* = 4, D. S*=6	
9	6	M4D6-P6	M.S* = 4, D. S*=6	
10	0	M5D4-P	M.S* = 5, D. S*=4	
11	3	M5D4-P3	M.S* = 5, D. S*=4	
12	6	M5D4-P6	M.S* = 5, D. S*=4	

M.S*: main steel, D.S*: Distribution steel.

### Materials

**Fig. 3:**
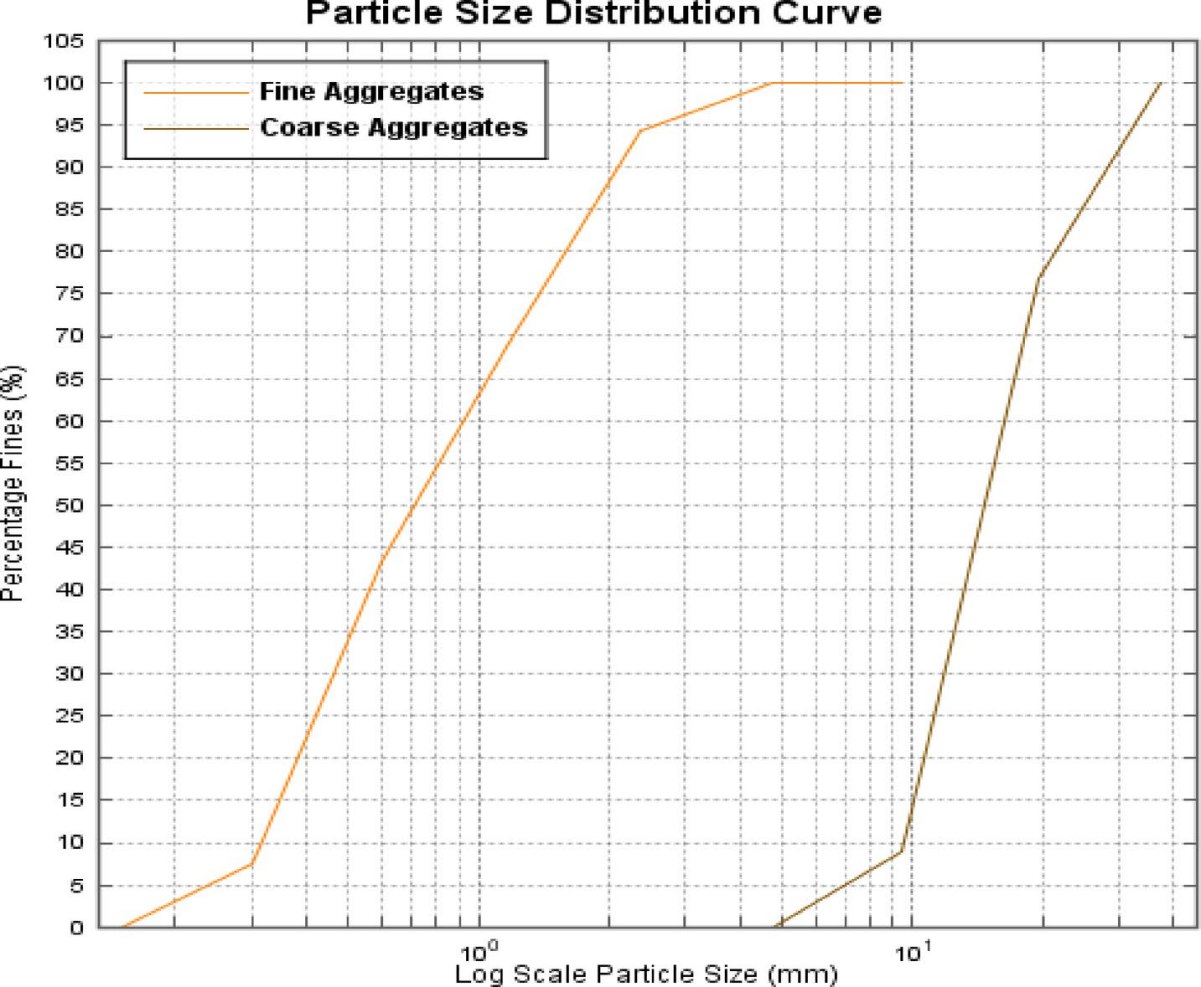
Fine and coarse aggregate particle size.

Type I ordinary Portland cement (OPC) by the ASTM standard C150^19^was used in the casting of beams. Locally available coarse and fine aggregates were used for casting. The fine aggregate with a Fineness Modulus of 2.81 was used. The gradation curve of aggregates used in the mix design is shown in Fig. 3. Physical properties, such as specific gravity, water absorption, and unit weight of the coarse and fine aggregates used are shown below in Table 2. Steel of grade 60 was used for reinforcement in the beams casted. The yield strength and ultimate strength of the steel were 450 MPa and 661 MPa respectively.

**Table 2:** Physical properties of coarse and fine aggregates.

	Apparent Specific gravity (kg/m^3^)	Absolute Specific gravity (kg/m^3^)	Water absorption (%)	Unit weight (kg/m3)		Density Ratio
				Loose	Compacted	
Coarse aggregate	2.64	2.64	0.51	1360	1522	0.90
**Fine aggregate**	2.63	2.60	2	1410	1650	0.854

**Table 2.1:** Mix design of concrete.

Cement	Sand	Coarse Aggregate	Water
kg/m^3^	kg/m^3^	kg/m^3^	kg/m^3^
370	680	1370	145

### Thermophyscial properties of plaster

One of the objectives of this study was to evaluate the effect of plaster as an insulation material under fire exposure. For this purpose, a plaster of 1:6 was considered in this study to plaster the beams before fire exposure. This mix composition was considered as sand has less thermal conductivity than cement. The plaster mix develop as insulation material had dry density of 1677 kg/m^3^, with thermal conductivity of 1.80 W/m·K and specific heat of 0.85 kJ/kg·K at room temperature.

#### Fire setup

Most of the research has conducted fire experiments in controlled environments such as electric furnaces or furnaces with burners, which do not provide much real fire exposure. To achieve a real-time fire scenario, the cast beams were exposed to fire in an open environment with air circulation. Unlike the electric furnaces, where the temperature is maintained at a fixed level, the temperature in this case was not constant but ranged between 600℃ and 800℃. A thermometer monitored the temperature, with a maximum reading of 800℃. Based on the simplified approach and the global response of uncontrolled fire thermal gradient analysis was not considered instead as per Eurocode (EN 1992-1-2) a uniform distribution of the temperature was considered across the cross-section of beam. The beams were subjected to fire for durations of 3 h and 6 h for all specimens, including the control beams. Duration of 3 h and 6 h were selected based on Eurocode (EN 1991-1-2) as it specifies the 3 h fire duration for critical high structure members and 6 h duration for ultra-high fire-resistant structures such as nuclear facilities, tunnels etc.

**Fig. 4:**
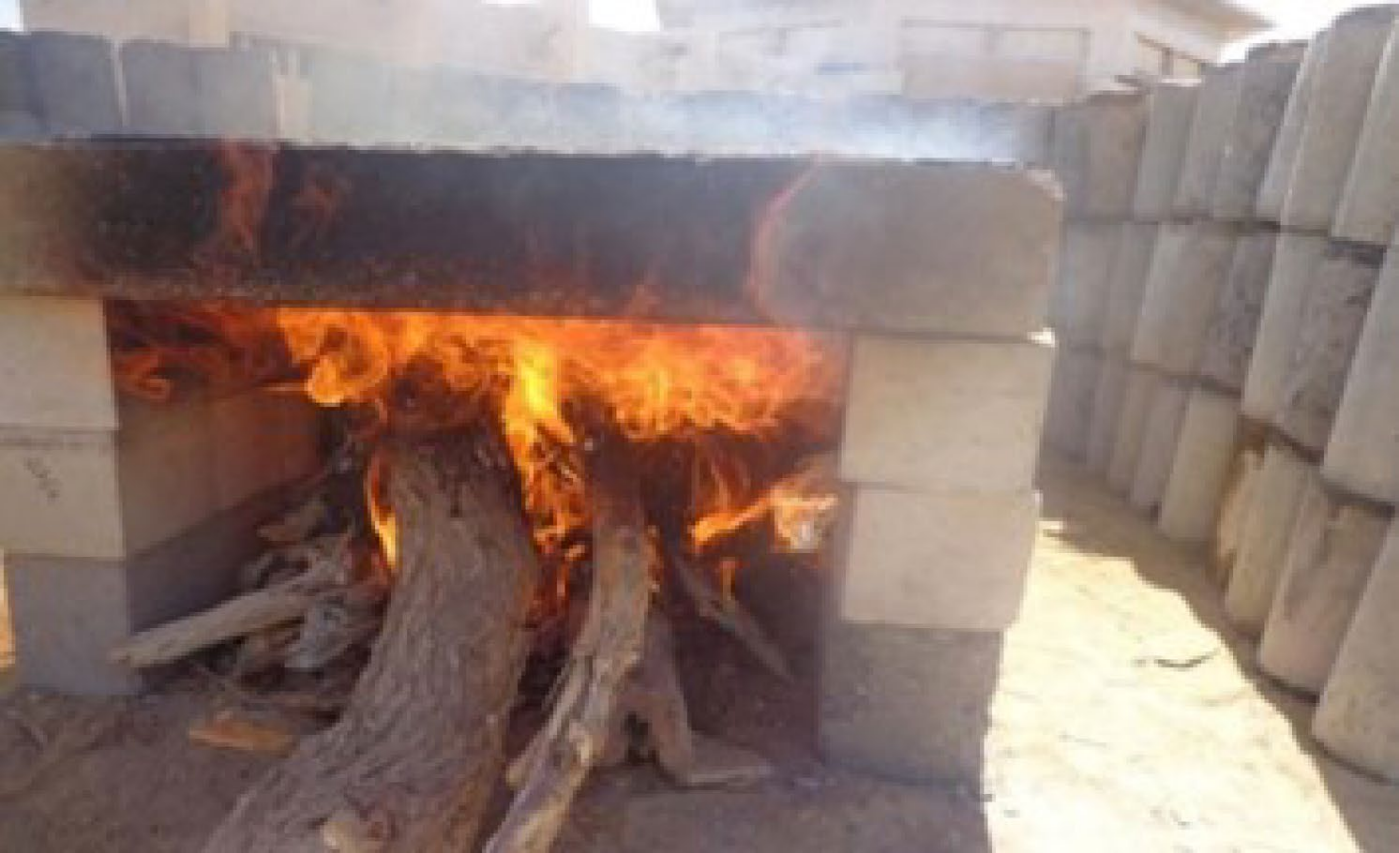
Beams under fire during experiment.

The beams were simply supported on an open furnace made of concrete cubes, and to maintain the above-mentioned temperature range, the furnace was closed from three sides with a 450 mm high concrete cylinder wall. No external loading was applied during the fire exposure to the beams. Figure 4 shows the beams exposed to fire during the experiment.

### Flexural testing

In Fig. 4, the beams are shown to have turned black due to the fire. After the fire, the beams were left to cool in the open air for 24 h. Subsequently, the beams were washed and painted white before proceeding to testing to obtain more details about the cracking patterns and failure modes under the three-point loading test. A three-point loading test was performed on the beams after the fire to understand the post-fire behavior of reinforced concrete beams. The three-point loading test was conducted on a Universal Testing Machine (UTM) with a capacity of 1800 kN. An initial load of 5 kN was set to experiment. The cracking patterns and failure modes of all the specimens are discussed below.

## Results and discussions

Prepared beams after exposure to fire for 3 h. and 6 h. were subjected to flexural testing by a three-point loading method. The considerable outputs were load carrying capacity curve, cracking behavior, failure modes and the residual moment capacity of the beams after and before fire exposure.

### Physical Inspection

**Fig. 5:**
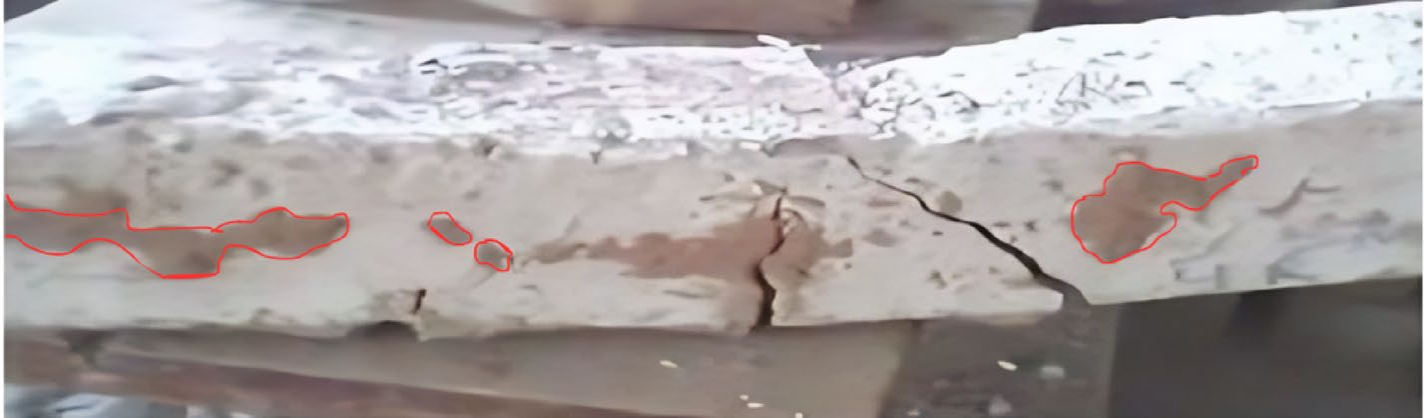
Spalling of beams after fire exposure.

After the fire beams had cooled in the open air, all specimens exposed to the fire were visually inspected for major failures. However, none of the specimens subjected to fire showed any significant cracks or severe damage. Some samples exhibited spalling due to excessive dehydration from the high temperatures, as shown in Fig. 5. The possible reasons for the spalling may include the effects of dehydration, as these specimens could have been subjected to a maximum temperature of 800℃.

### Cracking pattern and failure type

**Fig. 6:**
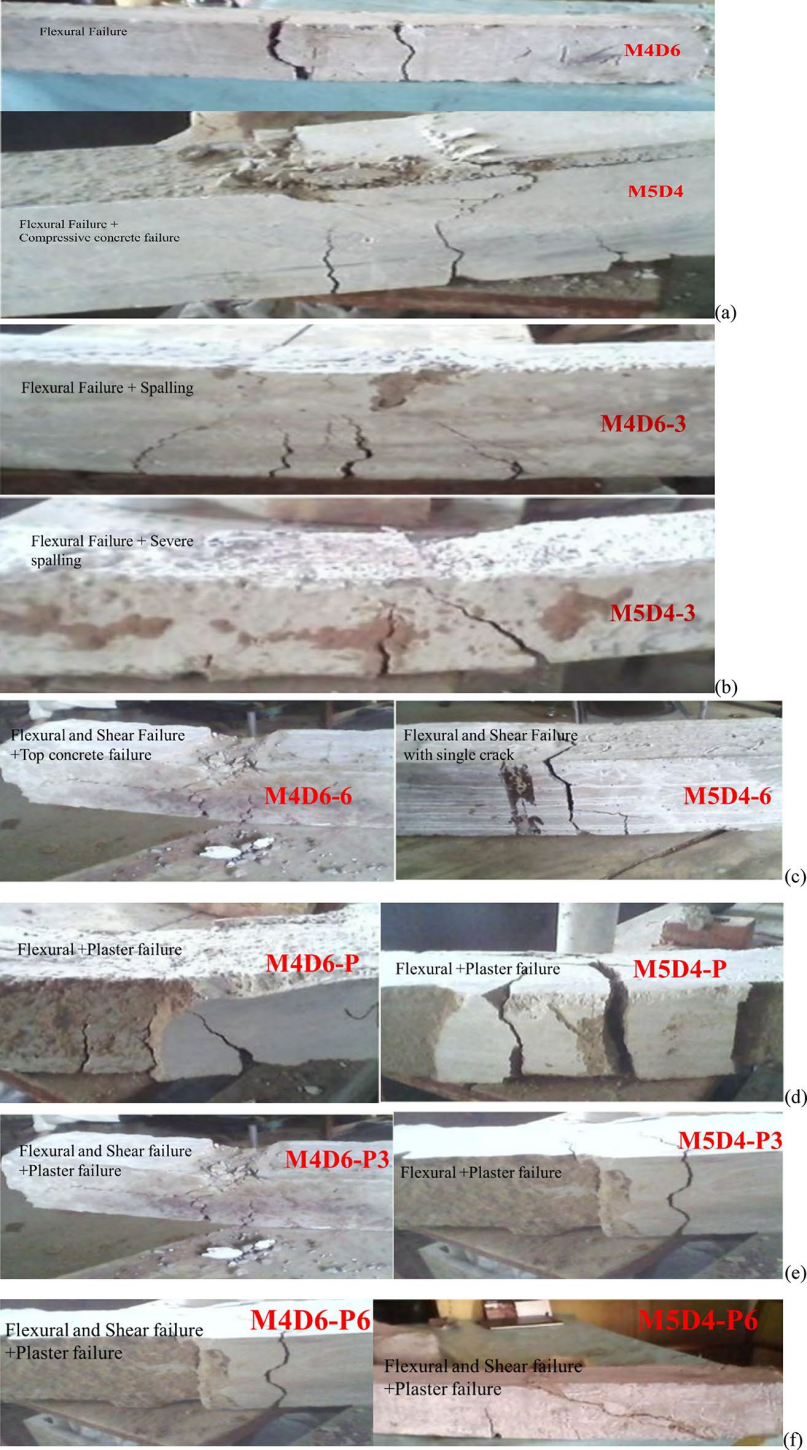
Cracking pattern and failure modes in reinforced beams (**a**) Control samples, (**b**) After 3 h. of fire exposure, (**c**) After 6 h. of fire exposure, (**d**) Plaster beams failure before fire exposure, (**e**),(**f**) failure of plaster beams after 3 h. and 6 h. of fire exposure.

Figure 6 below shows the cracking pattern and failure modes of the control samples as well as the specimen exposed to fire for 3 h. and 6 h. Flexural and shear-type failure can be observed in Fig. 6 for the specimen studied. Both M4D6 and M5D4 showed flexural failure however, the M4D6 beam showed complete failure at the peak load while M5D4 showed severe cracks but not a brittle failure. Additionally, M5D4 showed an over-reinforced failure, this could be due to the addition of extra rebar in the tension zone. M4D6-3 and M5D4-3 failed in flexural mode, but the former showed more cracks in the tension zone as compared to M5D4-3. A bit different pattern in the failure modes of M4D6-6 and M5D4-6 was observed compared to other samples. M4D6-6 showed a mixed flexural shear behavior with severe damage at the top and bottom corners. On the contrary, M5D4-6 showed a complete flexural failure with just a single crack propagating throughout the depth and width of the beam. It can be observed that shear stirrups and additional reinforcement didn’t make any difference in the failure pattern after 3 h. and 6 h. of fire exposure; similar behavior is also reported by Liu et al.^20^. Similarly, M4D6-P and M5D4-P showed flexural failure followed by the plaster failure of the beams. As it can be seen, the M5D4-P showed a brittle failure as compared to M4D6-P. M4D6-P and M5D4-P, after fire exposure, have shown flexural and shear failure with the complete failure of plaster as well.

### Load-deflection behavior

**Fig. 7:**
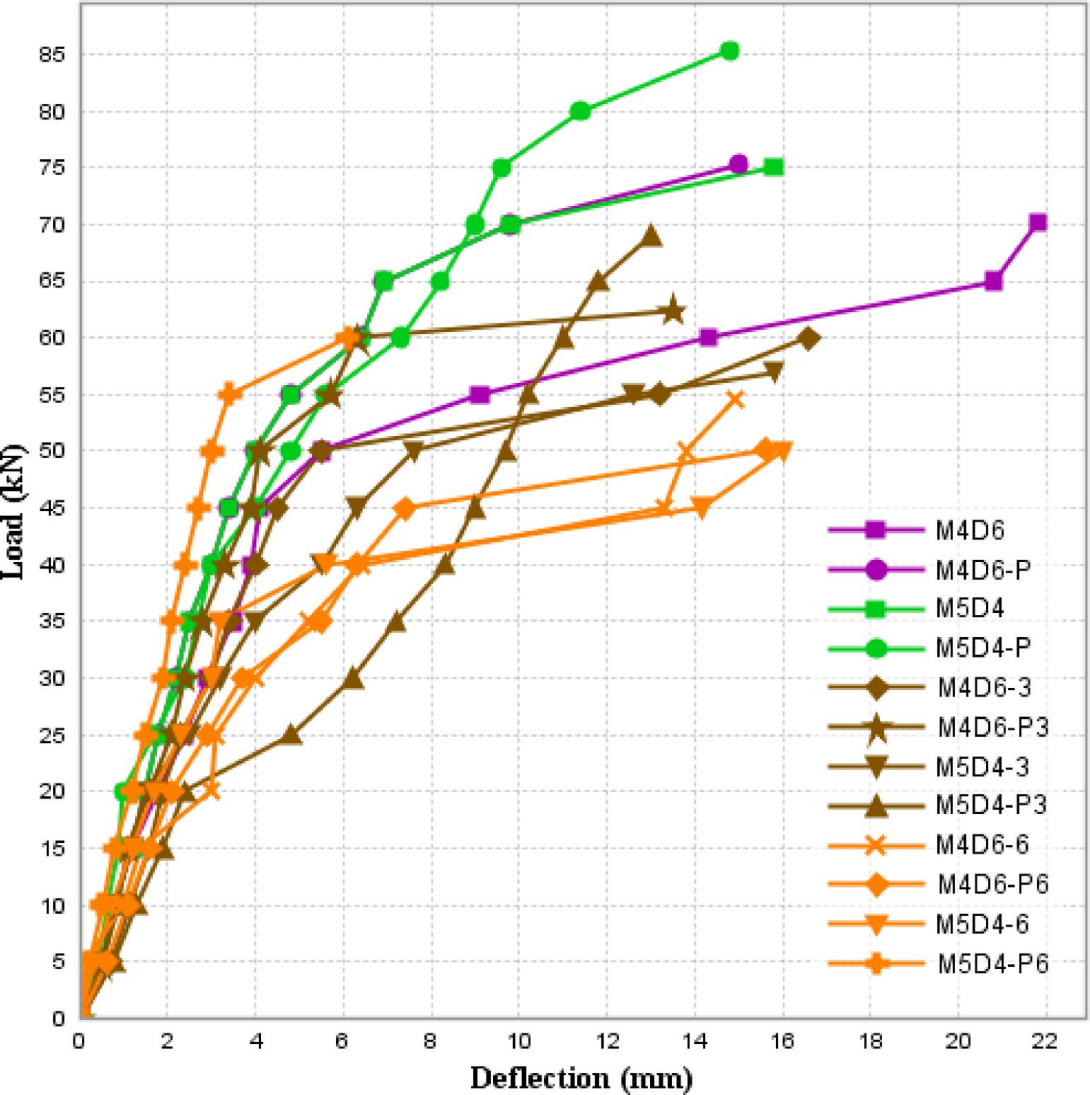
Load deflection curve of beams exposed to fire.

This study investigates the effects of fire exposure on the peak load, deflection, and strength loss of reinforced concrete beams, both with and without plaster protection. The table presents data on beams exposed to fire for 3 and 6 h. The load-deflection curve of the beams subjected to fire for varying durations is illustrated in Fig. 7 below. The initial phase is the elastic stage, where the load-deflection curves displayed an almost linear relationship. This was followed by the elastic-plastic stage, in which the curve’s slope began to decrease compared to the elastic phase, resulting in a nonlinear load-deflection behavior. Finally, in the failure stage, the load increased only gradually, even as the deflection continued to rise. Table 3 below shows the percentage decrease in the load-carrying capacity of beams subjected to fire compared to the control samples.

### Peak load analysis

For M4D6, the peak load before fire exposure was 68.29 kN. After 3 h of fire exposure (M4D6-3), the peak load dropped by 14% to 58.26 kN. After 6 h (M4D6-6), the load further decreased by 33.9%, reaching 50.98 kN. M5D4 initially had a higher peak load of 74.6 kN. After 3 h of fire exposure (M5D4-3), it fell by 23% to 57.4 kN, and after 6 h (M5D4-6), the peak load decreased to 50.75 kN, a 31.9% reduction. Plastered beams demonstrated greater resistance to fire exposure. For M4D6-P, the initial peak load was 75.33 kN. After 3 h (M4D6-P3), the load decreased by 17.1% to 62.4 kN, and after 6 h (M4D6-P6), it fell by 33.45% to 50.13 kN. M5D4-P showed an initial peak load of 85.3 kN, the highest among the specimens. After 3 h (M5D4-P3), it dropped to 69 kN, indicating a 19.1% reduction. After 6 h (M5D4-P6), the peak load was reduced to 60 kN, representing a 29.6% loss.

### Deflection and strength loss

The specimen subjected to fire for 3 h. as well as for 6 h, showed a decreasing trend in load-carrying capacity, as is evident from other studies^20–22^, [24] that strength decreases with fire exposure. The deflection of M4D6 decreased from 17.8 mm (no fire) to 12.8 mm (M4D6-3) after 3 h, demonstrating a considerable reduction in ductility. However, after 6 h (M4D6-6), the deflection increased slightly to 13.6 mm. M5D4 experienced a slight increase in deflection after 3 h (from 12.2 mm to 13.9 mm) but then reduced to 13.2 mm after 6 h. The deflection behavior of plastered beams was different. M4D6-P showed a 2.8 mm reduction in deflection after 3 h (M4D6-P3). However, after 6 h (M4D6-P6), the deflection increased to 15.6 mm. M5D4-P followed a similar trend, showing a significant drop in deflection from 14.8 mm to 6.1 mm after 6 h (M5D4-P6), indicating a sharp reduction in ductility. Plastered beams exhibited greater resilience to strength loss. The maximum strength loss in unplastered beams was 33.9% (M4D6-6), while the maximum strength loss in plastered beams was slightly lower at 33.45% (M4D6-P6). For M5D4 beams, plastering reduced the strength loss from 31.9% (M5D4-6) to 29.6% (M5D4-P6). (Due to technical issues, the search service is temporarily unavailable.) The unexpected increase in deflection at failure for beams M4D6-6 and M5D4-6 after 6 h of fire exposure, despite their reduced flexural stiffness, can be due to several interrelated mechanisms. Prolonged fire exposure causes extensive microcracking within the concrete matrix, which redistributes stresses more uniformly across the beam rather than localizing at critical points. This distributed damage allows the beam to undergo greater deformation before ultimate failure, even as stiffness diminishes. Additionally, thermal creep in both concrete and steel reinforcement at sustained high temperatures (700^o^C) contributes to time-dependent deformation, which enable the beam to deflect further under load over time. The second factor is the cooling phase post-exposure of fire which might have introduced residual stresses and produces more cracks, which resulted in modifying the load-deflection response. The similar effect has been noticed by the Engen et al. [25]. The results indicate that plastered beams have a higher initial load capacity and greater resistance to strength loss compared to unplastered beams. Plaster acts as an insulating layer, delaying heat transfer to the reinforcement and concrete, which enhances the fire performance of the beams. However, after prolonged exposure (6 h), both plastered and unplastered beams experience significant strength loss. The deflection behavior highlights that plastered beams retain more ductility after 6 h of fire exposure. Nevertheless, in the case of M5D4-P6, the deflection drops significantly, which could indicate localized failure or spalling.

**Table 3:** Strength loss of beams exposed to fire for a duration of 3 h. And 6 h.

Sr. #	Specimen	Peak Load () (kN)	Strength loss (%)	Ultimate Deflection ($\:{\varvec{\varDelta\:}}_{\varvec{u}}$) (mm)	Yield Deflection $\:{\varvec{\varDelta\:}}_{\varvec{y}}$ (mm)	Ductility Ratio $\:\frac{{\varvec{\varDelta\:}}_{\varvec{u}}}{{\varvec{\varDelta\:}}_{\varvec{y}}}$
1	M4D6	68.29	-	22.0	4.5	4.888889
2	M4D6-3	58.26	14	16.2	3.8	4.263158
3	M4D6-6	50.98	33.9	15.0	4.0	3.750000
4	M5D4	74.6	-	15.8	3.9	4.051282
5	M5D4-3	57.4	23	15.8	4.2	3.761905
6	M5D4-6	50.75	31.9	15.0	4.0	3.750000
7	M4D6-P	75.33	-	15.0	4.2	3.571429
8	M4D6-P3	62.4	17.1	13.5	3.9	3.461538
9	M4D6-P6	50.13	33.45	15.6	4.5	3.466667
10	M5D4-P	85.3	-	14.8	4.4	3.363636
11	M5D4-P3	69	19.1	15.8	3.8	4.157895
12	M5D4-P6	60	29.6	6.1	2.2	2.772727

### Flexural stiffness (EI) of fire exposed beams

The flexural stiffness of the reinforced concrete beams exposed to fire is crucial parameter to discuss as the level of damage after fire can be very much analyzed by computing flexural stiffness. Fire exposure significantly degrades the mechanical properties of concrete and steel, leading to a reduction in the flexural stiffness of reinforced concrete (RC) beams. The flexural stiffness of the plaster and unplaster beam exposed to fire was calculated by the Eq. (1) below.

1 $$\:EI=\frac{P.{L}^{3}}{48.\varDelta\:}$$

Where $\:P$ is the load and $\:L$ is the span length of 815 mm and Δ is the deflection corresponding to initial load.

The calculated flexural stiffness for the beams exposed to fire for 3 h. and 6 h. is tabulated Table 4.

**Table 4:** Flexural stiffness of the plaster and unplaster beams exposed to fire.

Specimen	Peak Load (kN)	10% Load (*P*, kN)	Deflection at 10% Load (Δ, mm)	Flexural Stiffness EI (×10⁹ *N*·mm²)
M4D6	68.29	6.83	0.8	9.23
M4D6-3	58.26	5.83	1.0	6.62
M4D6-6	50.98	5.10	1.2	5.52
M5D4	74.60	7.46	0.7	12.49
M5D4-3	57.40	5.74	0.9	7.71
M5D4-6	50.75	5.08	1.1	6.37
M4D6-P	75.33	7.53	0.6	14.38
M4D6-P3	62.40	6.24	0.8	9.05
M4D6-P6	50.13	5.01	1.0	6.48
M5D4-P	85.30	8.53	0.5	19.70
M5D4-P3	69.00	6.90	0.7	12.16
M5D4-P6	60.00	6.00	0.9	8.32

The flexural stiffness analysis reveals the effect of fire exposure to the reinforced concrete beam and the effect of plaster to overcome the damage degradation damage to beams after fire. For unplastered control beams, the initial stiffness values (9.23 × 10⁹ N·mm² for M4D6 and 12.49 × 10⁹ N·mm² for M5D4) align with expectations, where the higher stiffness of M5D4 reflects its additional tensile reinforcement. However, after 3–6 h of fire exposure, these beams lose 28–49% of their initial stiffness a trend consistent with prior studies on thermal damage (Eurocode 2, 2004). The degradation mechanisms are twofold. First, thermal microcracking reduces the concrete effective cross-sectional area, particularly in unplastered beams where direct heat flux accelerates damage. Second, steel reinforcement loses stiffness at temperatures above 500 °C, further compromising structural rigidity. Plastered beams, in contrast, retain 50–60% of their initial stiffness under identical conditions. The plaster layer in reinforced concrete beams act as dual agent: it acts as a thermal barrier, slowing heat transfer to the concrete core, and provides mechanical restraint to limit crack propagation [26].

### Ductility ratio

The ductility ratio (δu/δy ​) is the ratio of the deflection at failure (δu​) to the deflection at yield (δy​), representing structures capacity to undergo large deformation before failure. A higher ductility ratio indicates better energy absorption and deformation capacity. The ductility ratio of the beams exposed to fore for pro-long duration has been evaluated to consider the RC beams response towards fire. Table 3 shows the ductility ratio of the beams exposed to fire for 3 h and 6 h duration. The ductility ratio values indicate the post-fire deformation capacity of the beams. Specimens without plaster, such as M4D6 (4.89) and M5D4 (4.05), exhibit higher ductility compared to their plastered samples, such as M4D6-P (3.57) and M5D4-P (3.36). This suggests that the plastered beams experience lower deformation before failure, likely due to the insulation effects reducing thermal degradation. The exposure time to fire also affects ductility, as seen in beams exposed for 6 h (M4D6-6, M5D4-6), which show reduced ductility compared to the beams exposed to prolong fire for only 3 h (M4D6-3, M5D4-3). The lowest ductility ratio (2.77) is observed in M5D4-P6, indicating that prolonged fire exposure significantly reduces the beam’s capacity to undergo plastic deformation. This unexpected behaviour of the M5D4-P6 is due to the differential thermal differences and the local damage produced due to the long duration of fire which reduces the ultimate load capacity as well as the ductility. Though unplaster beams showed high ductility compared to the plaster beams however the plaster layer as insulation material for 3 h restricted the lower ductility of the beams as observed in the beams exposed to fire for 6 h duration.

### Flexural capacity of fire-exposed beams

The load deflection behavior along with the peak load and maximum deflection are discussed in Table 5. Experimental flexural moment capacity of fire exposed beams was taken as $\:{M}_{u}={P}_{u}\times\:l/4$ as beam were subjected to three-point loading creating maximum moment at the center of the beam. The experimental moment capacity was compared to the code-based residual moment capacity of the fire-exposed beams. Flexural responses of beams subjected to high temperatures from the European standard codes EN-1991-1-1 [27] were adopted to compare the experimental flexural response of beams exposed to fire. Equations Eqs^2] and [3^ from the selected code are provided below.

2 $$\:{M}_{u}={\eta\:f}_{cd}\left(\lambda\:a\right)b(d-\frac{\lambda\:a}{2})$$

Equation^1^. provides the residual moment capacity after the fire exposure. The equivalent compressive stress distribution parameters for concrete are represented by η = 1.0. In this context, $\:a$ denotes the depth of the concrete compression zone. The width of the beam is represented by $\:b$, and $\:d$ refer to the effective depth of the beam’s section. These parameters are critical in analyzing the stress behavior within the concrete under load.

### Calculation of influencing parameters after fire exposure

#### Effective depth and width

It is evident from Eq^1^ that the effective area of the beam plays a vital role in estimating the moment capacity of the reinforced beam. However, in this study, there is no loss in the effective area of the beam after being exposed to fire for 3 h and 6 h. The effective width and depth of the beam remained the same before and after the fire at 152 mm. In the case of plastered beams, although failure of the plaster has been observed under the three-point loading test, the effective depth and width of the plastered beams were taken as 172 mm (152 + 20 mm).

### Concrete strength reduction

**Fig. 8:**
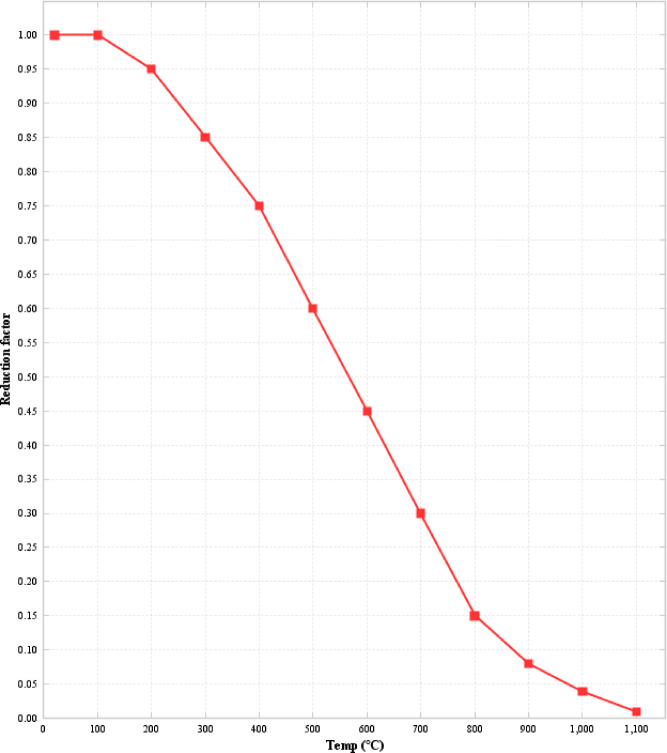
Compressive stress relationship for concrete at elevated temperatures [27].

During the experimental setup, the fire was configured to be uncontrolled, simulating a real fire scenario. As a result, it was not feasible to equip the beams with instruments to measure temperature variations over time; only the maximum temperature was recorded using asphalt temperature measuring thermometers. For analysis purposes, the standard EN 1991-1-1 strength reduction curve, shown in Fig. 8, is considered. The strength reduction is based on the equation provided below. Due to the lack of control over the fire and atmospheric conditions, the temperature ranged between 600 °C and 800 °C during the fire exposure for both 3 h and 6 h. In this study, a mean temperature of 700 °C was used to determine the strength reduction of the characteristic compressive strength of concrete, estimated to be 6 MPa as per Eq. 2.

3 $$\:\frac{3\epsilon\:{f}_{\text{c},\theta\:}}{{\epsilon\:}_{\text{c}1,\theta\:}\left(2+{\left(\frac{\epsilon\:}{{\epsilon\:}_{\text{c}1,\theta\:}}\right)}^{3}\right)}\:\:\:\:\:\:\:\:\:\:\:\:\:\:\:\:\:\:\:\:\:\:\:\:\:\:\:\:\:\:\:\:\:\:\:\:\:\:\:\:\epsilon\:\le\:{\epsilon\:}_{\text{c}1,\theta\:}$$

Linear or non-linear models can be used for the descending branch of the curve in the range

$$\:{\mathcal{E}}_{\text{c}\left(\theta\:\right)}<\mathcal{E}\le\:{\mathcal{E}}_{\text{c}\text{u}1,\theta\:}$$.

### Steel strength reduction

The tensile strength of steel is typically determined through tension tests on steel samples at room temperature. However, steel tensile strength decreases as the temperature rises when steel is subjected to fire. According to Eurocode 2, Part 1–2 [27], the reduction coefficient () is recommended to account for this decrease in strength during fire exposure, and it is used to assess the capacity of reinforced concrete (RC) members under fire conditions. After a fire, its strength can recover as the steel cools and returns to ambient temperature. Experimental studies, such as those by Lee [28], have shown that when steel is heated and then cooled, there is no significant reduction in yield strength if the temperature remains below 700 °C. In RC beams, the steel temperature is usually much lower than the fire’s gas temperature due to the protective cover of concrete. For plaster beam plaster as an insulation material the temperature within the steel reinforcement is generally lower than 700 °C and for the unplaster beam ignoring the steel strength reduction could slightly overestimate the flexural capacity of beams. However, for simplicity, the strength of steel in this study for both plastered and unplastered beams is assumed to fully recover after cooling, and therefore, a reduction coefficient () equals 1 was used. For ease of computation, has been used in the study.

### Moment capacity

**Fig. 9:**
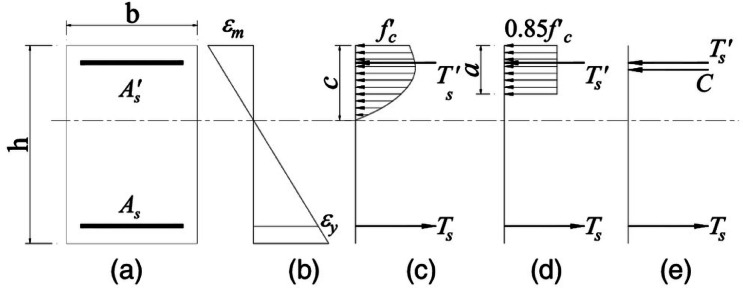
Doubly reinforced concrete section theoretical analysis (**a**) section details, (**b**) strain in section, (**c**) stress in tension and compression zone, (**d**) equivalent stresses and (**e**) forces [29].

Figures 9(a–c) show the cross-section, strain distribution, and stress distribution of a reinforced concrete beam when the tension steel reaches its yield point. Figure 9(d) illustrates the equivalent stress block, while Fig. 9(e) depicts the internal forces acting on the beam’s section. The force from the steel reinforcement in the tension zone () is given by Eq. (4), where the area of steel represents the tension steel, and denotes the stress in the steel. For the compression reinforcement, the corresponding compressive force (′) is expressed in Eq. (4), where ′ is the area of the compression steel. The equivalent stress block, modeling the compressive behavior of the concrete, has a depth of *a and* a stress of 0.85′. The equivalent compressive force exerted by concrete (C) is calculated using Eq. (5), where is the beam’s width, and f′ is the compressive strength of the concrete.

4 $$\:C+{T}_{s}^{{\prime\:}}={T}_{s}$$

5 $$\:0.85{f}_{c}^{{\prime\:}}ab+{A}_{s}^{{\prime\:}}{f}_{s}^{{\prime\:}}={A}_{s}{f}_{s}$$

6 $$\:a=\frac{{A}_{s}{f}_{s}-{A}_{s}^{{\prime\:}}{f}_{s}^{{\prime\:}}}{0.85{f}_{c}^{{\prime\:}}b}$$

The bending moment is calculated as discussed in Eq^1^.

### Comparison of Experimental and Analytical Results

The experimental moment capacity was calculated by considering a simple supported beam under a point load, as the testing procedure is similar to this. The experimental moment capacity was calculated by the Eq^7^ given below.

7 $$\:{M}_{u}=\frac{{P}_{u}\times\:L}{4}$$

The $\:{P}_{u}$ peak load is taken from Table 3 and $\:L$ is the effective span of the beam at 885 mm. For analytical purposes, the neutral axis depth depends on the residual compressive strength as well as the steel in both the tension and compression zones. Table 5 below provides details of all the parameters, including predicted moment capacity, neutral axis depth, and calculated moment capacity for both plastered and unplastered beams subjected to fire for durations of 3 h and 6 h.

**Table 5:** Experimental and numerical comparison of moment capacity of beam exposed to file.

Beam	Fire Duration (hrs)	Peak Load (kN)	$\:{\varvec{M}}_{\varvec{u}}$ Exp. (kNm)	Compressive Strength (MPa)	Neutral Axis Depth (mm)	$\:{\varvec{M}}_{\varvec{u}}\:$Ana. (kNm)	Percentage difference (%) of moment capacity
M4D6	0	68.29	15.10916	20	0	9.39	60.90695
M4D6-3	3	58.26	12.89003	6	0	9.39	37.27396
M4D6-6	6	50.98	11.27933	6	0	9.39	20.12061
M5D4	0	74.6	16.50525	20	47.68	13.29	24.193
M5D4-3	3	57.4	12.69975	6	47.68	11.44	11.0118
M5D4-6	6	50.75	11.22844	6	47.68	11.44	1.849323
M4D6-P	0	75.33	16.66676	20	0	10.87	53.32808
M4D6-P3	3	62.4	13.806	6	0	10.87	27.01012
M4D6-P6	6	50.13	11.09126	6	0	10.87	2.035534
M5D4-P	0	85.3	18.87263	20	12.64	15.6	20.97837
M5D4-P3	3	69	15.26625	6	42.13	13.96	9.357092
M5D4-P6	6	60	13.275	6	42.13	13.96	4.906877

The experimental and analytical moment capacities for beams exposed to fire, both plastered and non-plastered, reveal significant patterns. Significant variations exist between the experimental and analytical values for the non-plastered beams (M4D6 and M5D4), especially for the unexposed beams, with errors up to 60.9% for M4D6. The percentage differences decrease with increasing fire exposure (3 and 6 h), with M5D4-6 showing the lowest percentage difference at 1.85%. This alignment suggests that the analytical model represents the fire-damaged state of the beams more correctly. For fire-damaged beams, the closer alignment between experimental and analytical results suggests that the degradation effects of fire on material properties, including the loss in compressive strength and reinforcement yield strength, are reasonably well represented in the analytical model. Additionally, the post-fire residual strength of concrete and reinforcement may exhibit more predictable trends due to established empirical correlations for fire-damaged concrete. The influence of thermal strains, spalling, and concrete microcracking likely contributed to improved agreement between experimental and analytical results in fire-exposed specimens.

**Fig. 10:**
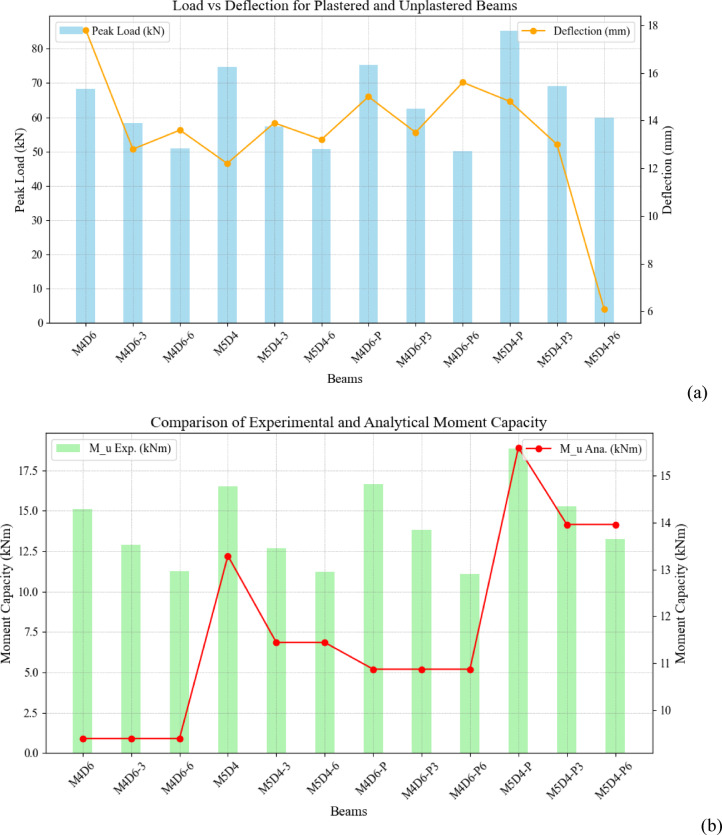
Comparison of peak load and moment capacity of doubly reinforced concrete beam against fire duration of 3 h. and 6 h., (**a**) load deflection behaviour of plastered and unplastered beams, (**b**) Experimental and Analytical moment capacity of plastered and unplastered beams.

Plastered beams (M4D6-P and M5D4-P) exhibit significant variations in the unexposed beams once more (up to 53.3% for M4D6-P). This might be because the plaster provides extra strength or insulation, which the analytical technique does not take into consideration. After exposure to fire, however, the differences become less pronounced, with M4D6-P6 and M5D4-P6 exhibiting negligible variances of 4.9% and 2.03%, respectively. The unexpected behavior of the M5D4-P6 could be due to spalling effect. Prolong fire exposure can develop some moisture, and which could result in explosive spalling at the concrete plaster interface [30]. Additionally, the thermal differential between plaster and concrete could weaken the mechanical properties of reinforced concrete beam which could be the possible reason for the reduced flexural strength of M5D4-P6 [31]. This implies that the fire-resistant properties of plaster produce more consistent and aligned performance between analytical and experimental outcomes. All things considered, the research shows that although the analytical models give cautious estimates, particularly for unexposed beams, their forecasts greatly improve in the event of a fire, especially for plastered specimens. The performance of plastered and unplastered beams in terms of peak load, deflection, and moment capacity are technically compared in Fig. 10. Plastered beams, as shown in Fig. 10(a), display larger peak loads and smaller deflections, suggesting stronger stiffness and load-bearing capability, in contrast to unplastered beams, which demonstrate weaker structural performance and greater flexibility under load. Plastered beams, namely M4D-P6 and M5D4-P, exhibit tighter alignment between the two in Fig. 10(b), which compares experimental and analytical moment capacities and suggests that the analytical model properly predicts their behavior. Unplastered beams, on the other hand, show larger disparities because experimental moment capacities do not match analytical expectations, most likely because of early cracking and flaws in the material that are not taken into consideration in the theoretical model. Plastering, in general, increases the load-bearing capacity and bending resistance of beams while minimizing deflection. The disparities between unplastered beam experimental and analytical results highlight the need for better models that account for defects in real-world construction and material attributes.

## Conclusion

This study considered various factors. The first is the fire scenario to be uncontrolled and natural like the real-life scenario, along with the effect of reinforcement in the tension zone as well as the shear reinforcement. The second is the same configuration of beams with a plaster of thickness 20 mm to understand the effect of plaster on the beams. The study concludes following.

- Effect of Fire on Peak Load: The peak load of both plastered and beams decreased significantly as the length of fire exposure increased. After 3 h and 6 h of fire exposure, M4D6 reduced the peak load in unplastered beams by 25.4% and 34.8%, respectively. In a similar vein, M5D4’s peak load dropped by 31.9% and 23.1%, respectively, following three and six hours of fire exposure. Conversely, plastered beams showed higher overall peak loads. For instance, M5D4-P had the highest peak load of 85.3 kN prior to fire exposure, while M4D6-P had a peak load of 75.33 kN, which was higher than the unplastered M4D6.
- Moment Capacity Degradation: The moment capacity (M_u_ Exp.) of the beams decreased significantly after being exposed to fire. After three hours and six hours, the moment capacity of unplastered M4D6 beams decreased by 14.7% and 25.4%, respectively. Less moment capacity reduction occurred in M5D4 (23% and 31.9%, respectively). Conversely, a larger proportion of the moment capacity of plastered beams was retained. Comparable exposure intervals resulted in M4D6-P losing 17.1% of its moment capacity after 3 h and 33.45% after 6 h, while M5D4-P lost just 19.1% and 29.6%.
- The Protective Function of Plaster and Its Effect on Moment Capacity: After being exposed to fire, plastered beams continuously outperformed unstained beams in terms of peak load and moment capacity retention. For example, the plastered M4D6-P kept 66.55% of its moment capacity after 6 h of fire exposure, but the unplastered M4D6 lost 63.65% of its moment capacity. The information shows that plaster significantly prevents fire-related deterioration, improving the beams’ overall structural performance. Furthermore, the percentage discrepancy between experimental and analytical moment capacities was considerably reduced in plastered beams. After six hours of fire exposure, M5D4-P6, for instance, only shown a 4.9% difference, but the unplastered M4D6-6 had a significantly greater 20.12% difference.
- No significant differences in crack patterns, failure progression, and load-displacement response between beams with and without stirrups were observed in this study which suggests that shear was not the governing failure mode. This could be due to that fact that beams had a shear span-to-depth ratio (a/d) within the range < 4 where flexural failure is expected to dominate, hence the role of stirrups in enhancing shear resistance was less pronounced.
- Design Evaluation: A simple design approach was used to evaluate the moment capacity of doubly reinforced concrete beams exposed to fire. This approach could help in the post-fire analysis of reinforced concrete beams. The analysis from the graphs suggests that including plaster in the design adds a protective layer, significantly improving the load-bearing capacity and resistance to deflection, making it a critical factor in post-fire performance.

### Limitations and recommendations

The study covers the global response of plaster and unplaster reinforced concrete beams subjected to uncontrolled fire as per the simplified approach. The study doesn’t consider the thermal gradient analysis as well the pre-loading effect to replicate the real-life scenario. For a more detail and better results a thermal gradient analysis and pre-loading effect is suggested in the future.

## Data Availability

All the data considered in the study has been published in the manuscript.
